# Focus on POCUS: Identification of Early Successful Intubation by Point-of-Care Ultrasound Versus End-Tidal Carbon Dioxide: A Prospective Comparative Study

**DOI:** 10.4274/TJAR.2024.241720

**Published:** 2024-12-16

**Authors:** Soma Ganesh Raja Neethirajan, Ganeshamoorthy Baskar, Aruna Parameswari

**Affiliations:** 1 Sri Ramachandra Institute of Higher Education and Research Department of Anaesthesiology and Pain Medicine, Chennai, Tamilnadu, India

**Keywords:** Airway management, end-tidal carbon-di-oxide, endotracheal intubation, intubation, POCUS

## Abstract

**Objective:**

Successful endotracheal intubation is a key step in advanced airway management. The gold standard confirmation for successful endotracheal intubation is end-tidal carbon dioxide (etCO_2_) monitoring, although recent studies suggest that ultrasound can also be used. In this study, we explored the time-sensitive early recognition of successful endotracheal intubation by comparing ultrasound and etCO_2_ monitoring.

**Methods:**

The study included 104 patients who were posted for elective surgery under general anaesthesia requiring endotracheal intubation. The time from removal of the face mask to ultrasound visualization of flutter in the trachea was compared with that of the appearance of six consecutive capnography waveforms following endotracheal intubation.

**Results:**

Ultrasound was a faster tool for recognizing successful endotracheal intubation [(21.63±7.38) seconds] compared with capnography [(40.62±7.93) seconds].

**Conclusion:**

eCO_2_ requires more time for 6 continuous waveforms to confirm successful intubation and has a false positive rate. Supplementing the gold standard etCO_2_ with ultrasound is faster and reliable in patients with low pulmonary blood flow without needing positive pressure ventilation, such as during cardiopulmonary resuscitation, in high-risk emergency intubations, such as in trauma, or in difficult airways where intubation can be confirmed in real time. Ultrasound is a reliable and faster tool for the early identification of successful endotracheal intubation than end-tidal carbon dioxide.

**Main Points**

• End-tidal carbon dioxide (etCO_2_) requires more time for 6 continuous waveforms to confirm successful intubation and has a false positive rate.

• Supplementing etCO_2_ with ultrasound is faster and more reliable, especially in patients with low pulmonary blood flow who do not need positive pressure ventilation, such as during cardiopulmonary resuscitation, in high-risk emergency intubation, such as in trauma, or in difficult airway situations where intubation can be confirmed in real time.

• Ultrasound is a reliable, rapid, and valuable tool for the early identification of successful endotracheal intubation.

## Introduction

The key step in advanced airway management is endotracheal intubation, which is performed to maintain ventilation and to deliver anaesthetic gases under general anaesthesia. Unintentional esophageal intubation (which is around 2.7 to 25%^1, 2^) dislodgement, and misplacement of the tube are potential catastrophic complications during intubation that result in rapid clinical deterioration of the patient causing hypoxemia, hemodynamic instability, and death.^3^

The confirmation of successful endotracheal intubation is usually performed by direct visualization of the tube entering the glottic opening, chest auscultation, bilateral chest movement, fogging of the endotracheal tube (ETT), capnography waveform, and radiological means (such as ultrasound and X-ray). End tidal carbon dioxide (etCO_2_) is the gold standard for identifying successful endotracheal intubation with 100% sensitivity and 100% specificity.^4, 5^ Recent studies suggest that ultrasound can be used to confirm endotracheal intubation and has equal validity as etCO_2_ for confirming successful endotracheal intubation.^6, 7, 8, 9^

In this prospective observational study, we compared ultrasound and end-tidal carbon dioxide for the early recognition of successful endotracheal intubation.

## Methods

After Sri Ramachandra Institute of Higher Education and Research, Institutional Research Ethics Committee approval (approval no.: EC/NEW/INST/2023/TN/0320, date: March 12, 2024), a prospective, single center, observational study was conducted at our tertiary care hospital. The study included 104 patients aged 18-75 years who underwent general anaesthesia requiring endotracheal intubation. Patients with expected difficult laryngoscopy, indication for awake fiber-optic intubation, parturient, and refusal to participate in the study were excluded from the study.

**Figure 1:**
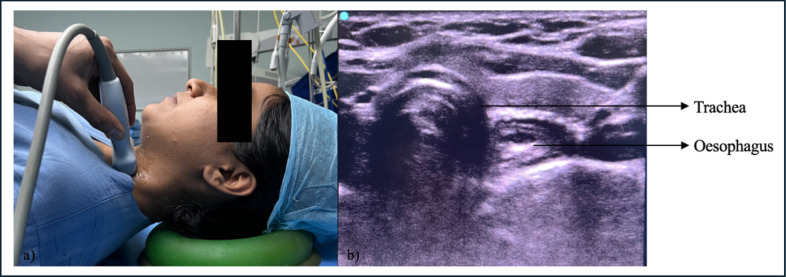
a) Scanning technique to visualize the trachea and esophagus. b) ultrasound image showing the trachea and esophagus in the same field

**Figure 2:**
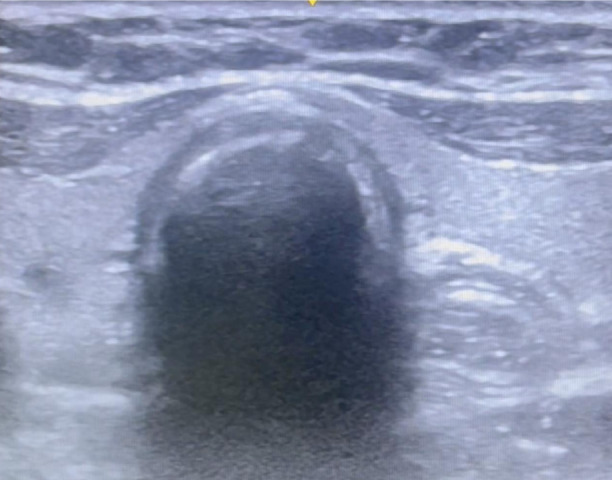
Ultrasound image of endotracheal intubation-bullet sign

**Figure 3:**
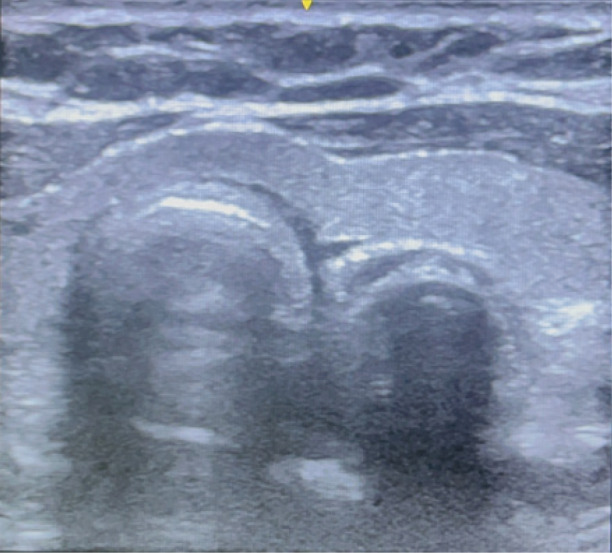
Ultrasound image of esophageal intubation showing the double bubble sign

After written informed consent, the patient was wheeled inside the operating room, and baseline monitors were connected and pre-oxygenated for 5 min with 100% oxygen. The patient was intravenously administered fentanyl (2 µg kg^-1^ and propofol 2 mg kg^-1^ intravenously, and paralyzed with vecuronium (0.1 mg kg^-1^). An ultrasound probe (HFL38, 13-6 MHz Linear transducer, Edge II, Fujifilm Sonosite Inc, Bothell, USA) was placed over the anterolateral aspect of the neck on the left side, at the level just below the cricoid cartilage, to visualize both the trachea and esophagus in the same field (Figure 1). The ultrasound was performed by a senior anaesthesiologist having expertise in airway ultrasound and doing it for more than 10 years. After 3 min. of mask ventilation, laryngoscopy and endotracheal intubation were performed. The timer was switched on, and the time from the removal of the face mask to the recognition of ETT in ultrasound entering trachea was noted. Similarly, the time from removal of the face mask to six square wave capnography was noted. Successful endotracheal intubation is identified by the bullet sign on ultrasound and the flutter created by the ETT inside the trachea, obliterating the reverberation artifact created by the tracheal cartilage (Figure 2). Esophageal intubation is identified by the ETT entering the esophagus, visualized in ultrasound as a double bubble sign with ETT inside esophagus by the side of trachea (Figure 3).

### Statistical Analysis

Sample size was calculated by taking the standard deviation (SD) of the time taken by ultrasonography to determine endotracheal intubation as 15.14 s according to the study by Chowdhury et al.^9^. The margin of error was estimated to be less than 3 s for the time taken by ultrasonography to determine endotracheal intubation. The other parameter considered for sample size calculation was 5% two-sided alpha error. The following formula was used to calculate the sample size:

Sample size (N) = ((Z_α/2_)^2^×SD^2^) ÷ d^2^

Where,

• SD = Standard deviation of the previous study.

• Z_α/2 _= Z_0.05/2 _= 1.96 (From Z table) at 5% alpha error.

• d = Estimated margin of error; and

Sample size (N) = ((1.96)^2^×15.14^2^) ÷ 3^2^ = 880.57 ÷ 9 = 97.84 ~ 98

According to the above calculation, the required sample size is 98.

When adding 10% non-response rate:

N* = N ÷ (1-0.1) = 98 ÷ 0.9 = 103.2 ~ 103/104.

Hence, the required sample size was 104.

Pescriptive analysis was performed at frequency and proportion for categorical variables. Continuous variables are presented as mean ± SD. Spearman’s rho correlation coefficient was used to check the relationship between two continuous variables. The intraclass correlation coefficient was used to check the agreement between the two methods. *P* < 0.05 was considered statistically significant. RStudio Desktop Version 2023.03.0+386 was used for statistical analysis. (Reference: RStudio Team (2023). RStudio: Integrated Development for R. RStudio, PBC, Boston, MA URL http://www.rstudio.com/).

## Results

**Figure 4:**
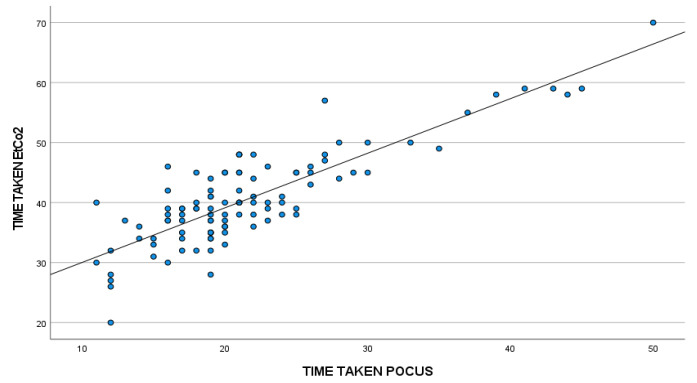
Scatter plot comparing time taken for POCUS vs. etCO_2_ etCO_2_, end-tidal carbon dioxide

A total of 104 patients were included in the study. The demographic data of all study participants (sex, age, height, weight, and body mass index) are presented in Tables 1 and 2, Figure 4.

The time taken by ultrasound for confirmation of endotracheal intubation was found to be 21.63±7.38 seconds, and the time taken for 6 waveform capnography was 40.62±7.93 seconds. The mean difference in recognition of successful endotracheal intubation between ultrasound and end-tidal carbon dioxide was 18.98±4.28 seconds, with ultrasound being early in recognition of successful endotracheal intubation (*P *< 0.001) (Table 3).

The correlation between the time taken for ultrasound and the end-tidal carbon dioxide was studied using Spearman’s correlation coefficient. There was a strong positive correlation between Time taken for POCUS and etCO_2_ (*P *< 0.001). Therefore, POCUS detected endotracheal intubation much earlier than end-tidal carbon dioxide in most of the study population (Table 4).

## Discussion

The present study compared two methods, ultrasonography and etCO_2 _in early recognition of successful endotracheal intubation in 104 patients who were posted for elective surgery under general anaesthesia requiring endotracheal intubation. In our study, the mean time taken by ultrasound was 21.63±7.38 seconds and time taken for getting 6 waveform capnography was 40.62±7.93 seconds to confirm endotracheal intubation. Ultrasound recognized endotracheal intubation quicker than capnography with a mean difference of 18.98±4.28 seconds (*P* < 0.001).

Endotracheal intubation and its placement inside the trachea are time-sensitive procedures. The most serious complication during ETT placement is unintentional esophageal intubation, the incidence of which ranges from 2.7% to 25%.^2, 3^ Several methods have been employed to confirm the ETT position like visual confirmation of tube entering the glottis, chest auscultation, chest wall movement, fogging inside the tube, capnography by etCO_2_, esophageal detector devices, and radiologically by ultrasound and X-ray. Visualization of tube, fogging and chest auscultation are subjective and should be supplemented with a gold standard and rapid method to identify the correct placement of ETT.

Most of the above-mentioned methods have several limitations, like chest auscultation was normal in 48% of unintended esophageal intubation as described by Caplan et al.^10^ which is due to the transmission of esophageal and gastric sounds to the chest wall due to its close anatomical proximity, having high false positivity rate. Visualization of the tube entering the glottic opening is operator dependent and can be difficult in cases of difficult laryngoscopy wherein the glottic view is limited or it can be difficult due to the presence of secretions or blood in larynx.^11^ Fogging or condensation inside the ETT is also a not reliable predictor for successful endotracheal intubation as 83% of esophageal intubation in animal studies showed condensation inside the tube.^12^

Due to these limiting factors and low reliability, secondary adjuvant methods should be used for the proper identification of successful endotracheal intubation. Capnography by measuring etCO_2_ from expired CO_2_ remains the gold standard and is considered the most reliable indicator to confirm proper ETT placement and has been included as Class 1 recommendation by the American Heart Association since 2010.^13, 14, 15, 16^

Asai and Shingu^17^ in their observation reported a normal capnography waveform initially despite the ETT being in the esophagus. This can be explained by the pooling of expired carbon dioxide in pharynx.^17^ Similarly, in cases of cardiac arrest where pulmonary blood flow is reduced, even during administration of high-quality cardiopulmonary resuscitation, the capnographic waveform has high false-positive rates due to several limitations like false positive waveform when the ETT lies at the hypopharynx, accidentally during cardiac arrest. One of the major disadvantages of etCO2 is the need for positive pressure ventilation for confirmation, which can be detrimental when ETT “is” in the esophagus, causing gastric distension, aspiration or even rupture of the esophagus. In addition, during mask ventilation, the exhaled alveolar gas containing carbon dioxide enters the stomach and causes a false-positive capnography waveform during esophageal intubation. On subsequent breaths during esophageal intubation, carbon dioxide levels decrease, resulting in a decrease in etCO_2_. Hence, capnography requires at least 6 continuous waveform for confirmation of endotracheal intubations.^18, 19^

etCO_2_ is the gold standard for intubation detection, and it will continue to do so. There is a time lag for the detection of esophageal intubation by etCO_2_. In fact, the presence of the first few waveforms of etCO_2_ during esophageal intubation misguides the anaesthesiologist toward endotracheal intubation. In patients with difficult airway or low perfusion states, such as shock due to polytrauma or major obstetric hemorrhage, if the esophageal intubation is misconstrued as endotracheal intubation, the first and best intubation attempts as well as the precious time to secure the airway are lost. Moreover, in emergency situations like during cardio-pulmonary resuscitation, attempts at securing the airway can be chaotic, stressful, and time-consuming. In addition, poor circulation during cardiac arrest can cause delayed response in end-tidal carbon dioxide levels on the monitor. Moreover, the amplitude of the etCO_2_ waveform will be reduced, and the time for it to appear will be delayed. Our argument is that the use of point-of-care-ultrasound during such situations to confirm endotracheal intubation will be more reliable, specific, and faster.

Ultrasound has several advantages over etCO_2 _being faster and more reliable, even in conditions with low pulmonary blood flow. In addition, ultrasound does not require positive pressure ventilation.^19^ Our study showed that ultrasound is a faster tool for the early recognition of successful ETT placement. The pitfalls of using ultrasound include availability of ultrasound, training of personnel, and booting time. The availability of ultrasound in every operating theater complex has become easier with the advent of POCUS. Training for airway assessment requires expertise, whereas identification of the trachea and esophagus is easier even by novice trainees. It takes less than 10 minutes to train novice trainees to identify the trachea and esophagus. The booting time of the ultrasound that we used was less than 25 seconds, unlike capnography, which can take minutes negating the time constraints associated with the use of ultrasound.

Our study involved placing a linear ultrasound probe on the anterolateral part of the neck below the level of the cricoid, and the trachea was visualized in the midline as an inverted U-shaped structure. It is characterized by a hyperechoic air-mucosal interface with a reverberation artifact that is visible posteriorly. The peristaltic movements that the patient experiences after swallowing indicate the presence of the esophagus, which is located easily deep within the trachea on its left side. The ETT appears as a hyperechoic brilliant structure when it traverses through the trachea, which aids in its vision by causing a transient flutter and acoustic shadowing or comet-tail effects.^7, 20^ In case of accidental esophageal intubation, the ETT entering the esophagus shows double bubble sign.^21^

According to a study conducted by Abhishek et al.^8^, both etCO_2_ and ultrasound can be used for confirmation of ETT placement, and in their study etCO_2 _was quicker than ultrasound. Their results were different from our study because we used six continuous waveforms in capnography for confirmation of endotracheal intubation.

Chowdhury et al.^9^, in their study compared various parameters in confirmation of ETT placement on intubations done by novice anaesthesia practitioners and concluded that ultrasound was a faster tool among ultrasound and chest auscultation. This was in accordance with our study, and these results can be extrapolated to general practice.

In our study, ultrasound detected misdirected ETT entering the esophagus in two patients (who were eliminated from statistical analysis), which was corrected immediately without requiring another laryngoscopy, which is another added advantage of using ultrasound for confirmation of endotracheal intubation. This real-time ultrasound guidance for endotracheal intubation is of immense value, especially in difficult airway situations.

We conducted this study to identify early successful endotracheal intubation. Our study did not statistically address the early identification of esophageal intubation, although it is possible in a study with a large sample size. We believe that etCO_2_ is the gold standard for confirming endotracheal intubation, and ultrasound should be used as an adjunct for identifying endotracheal intubation much earlier.

## Conclusion

Ultrasound can be used as a reliable and faster tool for confirming successful endotracheal intubation than capnography using etCO_2_. Ultrasound can be a more useful supplement to etCO_2_, especially in high-stake environments, such as during anticipated or unanticipated difficult airway, emergency intubations during cardiopulmonary resuscitation, and poly trauma where pulmonary blood flow is reduced leading to poor etCO_2_ waveforms.

## Ethics

**Ethics Committee Approval:** The study was obtained from Sri Ramachandra Institute of Higher Education and Research, Institutional Research Ethics Committee approval (approval no.: EC/NEW/INST/2023/TN/0320, date: March 12, 2024).

**Informed Consent:** Written informed consent was obtained from the patient.

## Figures and Tables

**Table 1. Descriptive Analysis of Gender in the Study Population (n = 104):** 

**Gender**	**Frequency**	**Percentages**
Male	59	56.73%
Female	45	43.27%

**Table 2. Descriptive Analysis of Demographic Variables in the Study Population (n = 104):** 

**Parameter**	**Mean ± SD**	**Median**	**Minimum**	**Maximum**	**95% CI**	
					**Lower**	**Upper**
Age (years)	43.36±14.88	43.5	18	75	40.46	46.25
Weight (kg)	71.33±13.95	71	48	107	68.61	74.04
Height (cm)	163.22±9.6	162	143	190	161.35	165.09
BMI (kg/m^2^)	26.79±4.82	26.5	16.9	38.83	25.85	27.73

SD, standard deviation; BMI, body mass index; CI, confidence interval

**Table 3. Comparison of Time Courses Between POCUS and EtCO2 (n = 104):** 

**Variable**	**Mean ± SD**	**Mean difference**	**Median**	**Minimum**	**Maximum**	**95% CI**		**95% confidence interval (CI) for mean difference**	***P* value**
						**Lower**	**Upper**		
Time taken by the POCUS (in seconds)	21.63±7.38	18.98±4.28	20	11	50	20.20	23.07	18.15-19.81	<0.001
Time taken by etCO_2_ (in seconds)	40.62±7.93		39	20	70	39.07	42.16		

etCO_2_, end-tidal carbon dioxide; SD, standard deviation; CI, confidence interval

**Table 4. Correlation Between Time Taken for POCUS vs. etCO2:** 

**Correlation between**	**Spearman’s rho correlation (95% CI)**c	***P* value**
Time taken to calculate POCUS vs. etCO_2_	0.738 (0.632 to 0.816)	<0.001

etCO_2_, end-tidal carbon dioxide; CI, confidence interval
